# Increases in sensory noise predict attentional disruptions to audiovisual speech perception

**DOI:** 10.3389/fnhum.2022.1027335

**Published:** 2023-01-04

**Authors:** Victoria L. Fisher, Cassandra L. Dean, Claire S. Nave, Emma V. Parkins, Willa G. Kerkhoff, Leslie D. Kwakye

**Affiliations:** ^1^Department of Neuroscience, Oberlin College, Oberlin, OH, United States; ^2^Yale University School of Medicine and the Connecticut Mental Health Center, New Haven, CT, United States; ^3^Roche/Genentech Neurodevelopment & Psychiatry Teams Product Development, Neuroscience, South San Francisco, CA, United States; ^4^Neuroscience Graduate Program, University of Cincinnati, Cincinnati, OH, United States; ^5^Department of Neurobiology, University of Pittsburgh, Pittsburgh, PA, United States

**Keywords:** multisensory integration (MSI), attention, dual task, McGurk effect, perceptual load, audiovisual speech, sensory noise, neural mechanisms

## Abstract

We receive information about the world around us from multiple senses which combine in a process known as multisensory integration. Multisensory integration has been shown to be dependent on attention; however, the neural mechanisms underlying this effect are poorly understood. The current study investigates whether changes in sensory noise explain the effect of attention on multisensory integration and whether attentional modulations to multisensory integration occur via modality-specific mechanisms. A task based on the McGurk Illusion was used to measure multisensory integration while attention was manipulated via a concurrent auditory or visual task. Sensory noise was measured within modality based on variability in unisensory performance and was used to predict attentional changes to McGurk perception. Consistent with previous studies, reports of the McGurk illusion decreased when accompanied with a secondary task; however, this effect was stronger for the secondary visual (as opposed to auditory) task. While auditory noise was not influenced by either secondary task, visual noise increased with the addition of the secondary visual task specifically. Interestingly, visual noise accounted for significant variability in attentional disruptions to the McGurk illusion. Overall, these results strongly suggest that sensory noise may underlie attentional alterations to multisensory integration in a modality-specific manner. Future studies are needed to determine whether this finding generalizes to other types of multisensory integration and attentional manipulations. This line of research may inform future studies of attentional alterations to sensory processing in neurological disorders, such as Schizophrenia, Autism, and ADHD.

## 1. Introduction

The interactions between top-down cognitive processes and multisensory integration have been heavily investigated and shown to be intricate and multidirectional (Talsma et al., 2010; Cascio et al., 2016; Stevenson et al., 2017). Previous research using different methods to manipulate attention and measure multisensory integration has demonstrated that multisensory integration is lessened under high attentional demand and relies on the distribution of attention to all stimuli being integrated (Alsius et al., 2005, 2007; Talsma et al., 2007; Mozolic et al., 2008; Koelewijn et al., 2010; Tang et al., 2016; Gibney et al., 2017). Studies investigating the time point(s) during which attentional alterations influence multisensory processing have identified both early and late attentional effects (Talsma and Woldorff, 2005; Talsma et al., 2007; Mishra et al., 2010). Additionally, multiple areas such as the Superior Temporal Sulcus (STS), Superior Temporal Gyrus (STG), and extrastriate cortex have been identified as cortical loci of attentional changes to multisensory processing (Mishra and Gazzaley, 2012; Morís Fernández et al., 2015). Collectively, these studies suggest that attention alters multisensory processing at multiple time points and cortical sites throughout the sensory processing hierarchy.

The precise mechanisms by which attention alters multisensory integration remain unknown. Multisensory percepts are built through hierarchical processing within sensory systems, coherent activity across multiple cortical sites, and convergence onto heteromodal areas (for an extensive review see Engel et al., 2012). Alterations in attention may primarily disrupt multisensory integration by interfering with integrative processes such as synchronous oscillatory activity across cortical areas or processing of multisensory information within heteromodal areas (Senkowski et al., 2005; Schroeder et al., 2008; Koelewijn et al., 2010; Al-Aidroos et al., 2012; Friese et al., 2016). Attention and oscillatory synchrony have been shown to interact in a number of studies (Lakatos et al., 2008; Gomez-Ramirez et al., 2011; Keil et al., 2016), thus strengthening the possibility of this potential mechanism. Although there is convincing evidence for attentional changes to integrative processes, there is a strong likelihood that disruptions in unisensory processing may explain, in part, attentional alterations in multisensory integration. An extensive research literature clearly demonstrates that attention influences unisensory processing within each sensory modality (Woldorff et al., 1993; Mangun, 1995; Driver, 2001; Pessoa et al., 2003; Mitchell et al., 2007; Okamoto et al., 2007; Ling et al., 2009). Additionally, attention has been shown to improve the neural encoding of auditory speech in lower-order areas and to selectively encode attended speech in higher-order areas (Zion Golumbic E. et al., 2013; Zion Golumbic E. M. et al., 2013). Alterations in the reliability of unisensory components of multisensory stimuli have been clearly demonstrated to alter patterns of multisensory integration such that the brain more heavily weighs input from the modality providing the clearest information (Deneve and Pouget, 2004; Bobrowski et al., 2009; Burns and Blohm, 2010; Magnotti et al., 2013, 2020; Magnotti and Beauchamp, 2015, 2017; Noel et al., 2018a). Thus, disruptions in attention may result in increased neural variability during stimulus encoding (sensory noise) causing degraded unisensory representations to be integrated into altered multisensory perceptions. Few studies have directly assessed the impact of attention on sensory noise and multisensory integration (Schwartz et al., 2010; Odegaard et al., 2016); thus, more exploration is needed to determine whether attentional influences on multisensory integration may be explained by increases in sensory noise.

Psychophysical tasks utilizing multisensory illusions may be able to determine whether attentional alterations in multisensory integration are mediated by disruptions in modality-specific processing. Multisensory illusions which result from discrepancies in information across modalities are ideally suited for this type of experimental design because the strength of the illusion can be altered by changing the reliability of the component unisensory stimuli and these effects can be modeled by measuring the ratio of visual and auditory sensory noise (Körding et al., 2007; Magnotti and Beauchamp, 2017). The McGurk effect is a well-known illusion that has been used to study multisensory speech perception (Mcgurk and Macdonald, 1976) and the effects of attention on audiovisual speech integration. The strength of the McGurk effect has consistently been shown to decrease with increasing perceptual load in dual-task studies (Paré et al., 2003; Alsius et al., 2005, 2007, 2014; Soto-Faraco and Alsius, 2009; Gibney et al., 2017). Because audiovisual speech can be understood through its unisensory components and requires extensive processing of the speech signal prior to integration (Zion Golumbic E. et al., 2013; Zion Golumbic E. M. et al., 2013), there is a strong likelihood that attentional alterations in audiovisual speech integration may be explained by disruptions to the unisensory processing of speech information. Specifically, disruptions in the encoding of visual speech components would be expected to weaken the McGurk Effect while disruptions in the encoding of auditory speech components would strengthen the McGurk Effect.

In this study, we investigate attentional influences on early auditory and visual processing by examining modality-specific attentional changes to sensory noise. In two separate experiments, participants completed a McGurk task that included unisensory and congruent multisensory trials while concurrently completing a secondary auditory or visual task. Sensory noise was calculated from the variability in participants’ unisensory responses separately for the auditory and visual modalities. Multiple regression analysis (MRA) was then used to determine the impact of visual noise, auditory noise, and distractor modality on McGurk reports at baseline and changes in McGurk reports with increasing perceptual load. We predicted that increases in perceptual load would lead to decreases in the McGurk effect and increases in sensory noise within the same modality as the distractor. Additionally, we predicted that changes in McGurk reports with increasing load would be best predicted by changes in visual noise (as compared to changes in auditory noise).

## 2. Materials and methods

### 2.1. Participants

A total of 172 (120 Females, 18–44 years of age, mean age of 22) typically developing adults completed this study. 57 (38 Females, 18–36 years of age, mean age of 22) participants completed trials with auditory distractors and 138 (82 Females, 18–44 years of age, mean age of 22) participants completed trials with visual distractors. Data from some participants overlaps with data previously published in Gibney et al. (2017). Twenty-three (23) participants completed both experiments in separate sessions. Participants were excluded from final analysis if they did not complete at least four repetitions of every trial type (45) or did not have a total accuracy of at least 60% on the distractor task for the high load condition (12). Thus, 115 participants were included in the final analysis. Participants reported normal or corrected-to-normal hearing and vision and no prior history of seizures. Participants gave written informed consent and were compensated for their time. Study procedures were conducted under the guidelines of Helsinki and approved by the Oberlin College Institutional Review Board.

### 2.2. Experimental design overview

We employed a dual-task design to determine the effects of attention within a specific sensory modality on McGurk perceptions and on sensory noise within each modality. Similar dual task designs have been shown to reduce attentional capacity (Lavie et al., 2003; Stolte et al., 2014; Bonato et al., 2015). Participants completed a primary McGurk task concurrently with a secondary visual or auditory distractor task for which the level of visual or auditory perceptual load was modulated. Full methodology for both the primary McGurk task as well as the secondary distractor tasks has been previously published in Dean et al. (2017) and Gibney et al. (2017); however, we provide a brief overview of all tasks here. All study procedures were completed in a dimly lit, sound-attenuated room. Participants were monitored via closed-circuit cameras for safety and to ensure on-task behavior. All visual stimuli were presented on a 24” Asus VG 248 LCD monitor at a screen resolution of 1,920°×°1,080 with a refresh rate of 144 Hz at a viewing distance of 50 cm from the participant. All auditory stimuli were presented from Dual LU43PB speakers which were powered by a Lepas LP-2020AC 2-Ch digital amplifier and were located to the right and left of the participant. SuperLab 4.5 software was used for stimulus presentation and participant response collection. Participants indicated their responses on a Cedrus RB-834 response box, and responses were saved to a txt file.

### 2.3. McGurk task

**FIGURE 1:**
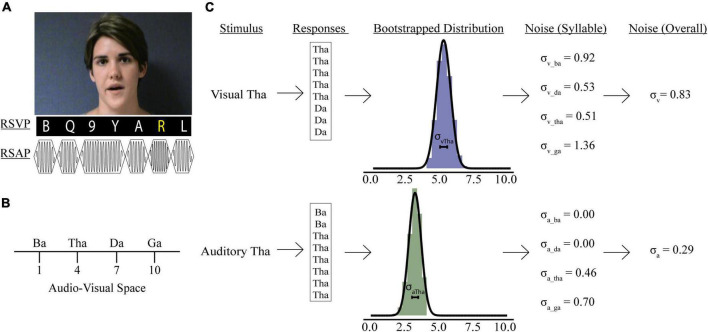
Psychophysics tasks and sensory noise calculations. **(A)** Participants watched videos of a woman speaking one of four syllables, after which they reported if she said: “ba,” “ga,” “da,” or “tha.” Rapid serial visual presentation (RSVP) or rapid serial auditory presentation (RSAP) stimuli accompanied speech videos during no load (NL), low load (LL), and high load (HL) blocks. For the visual distractor task, participants detected a yellow letter (LL) or a white number (HL). For the auditory distractor task, participants detected a high-pitched tone (LL) or a long-duration tone (HL). Identifiable human image used with permission. **(B)** Mapping of possible responses in representative audio-visual space. Panel **(C)** shows sensory noise calculations for an example participant. Sensory noise was calculated for each participant using responses from visual (top) and auditory (bottom) only trials. Gaussian distributions of these responses were determined via bootstrapping (middle), and the standard deviation of this distribution was calculated for each syllable. The overall visual (top, last panel) and auditory (bottom, last panel) noise for each participant was calculated as the average standard deviation of all syllabi within each modality.

Participants were presented with videos of a woman speaking one of four syllables “ba” (/ba/), “ga” (/ga/), “da” (/da/), or “tha” (/tha/, voiceless) (Figure 1A). Trials were either unisensory (visual-only; auditory-only) or multisensory (congruent; incongruent illusory; incongruent non-illusory). In unisensory trials, participants were presented with either the visual (visual-only) or auditory (auditory-only) components of the video for each syllable. Multisensory videos had both an auditory and a visual component and were either congruent (e.g., visual “ba” auditory “ba”), incongruent non-illusory (visual “ba” auditory “ga”), or incongruent illusory (visual “ga” auditory “ba”). Participants responded to the prompt, “What did she say?” by pushing one of four buttons labeled “ba,” “ga,” “da,” or “tha.” Although eye movements were not monitored, participants were explicitly instructed to maintain their gaze on the speaker’s mouth throughout the duration of the study. Each unisensory syllable was repeated 8 times for a total of 32 visual-only and 32 auditory-only trials. Each congruent multisensory syllable was repeated 8 times for a total of 32 total congruent multisensory trials. Lastly, there were 16 illusory incongruent and 16 non-illusory incongruent trials.

### 2.4. Secondary visual distractor task

Rapid serial visual presentation (RSVP) stimuli of white letters, yellow letters, and white numbers presented continuously below the McGurk videos (Figure 1A). Each letter and number in the RSVP stream was presented for 100 ms with 20 ms between letters and numbers. The visual distractor task included four condition types: distractor free (DF), no perceptual load (NL), low perceptual load (LL), and high perceptual load (HL). During distractor-free blocks, no visual or auditory distractors were presented; thus, participants completed the McGurk task in isolation. When the RSVP stream was presented concurrently with the McGurk task, participants were asked to either ignore it (NL), detect infrequent yellow letters (LL), or detect infrequent white numbers (HL). There was a 50% chance that the target would be present in each trial. After each presentation, participants were asked to respond first to the McGurk task then report whether they observed a target within the RSVP stream with a “yes” or “no” button press. Each load condition was completed in a separate block, and the order of blocks was randomized and counterbalanced across participants. Participants completed all perceptual load blocks in one session.

### 2.5. Secondary auditory distractor task

Stimuli consisted of rapid serial auditory presentation (RSAP) of musical notes at frequencies between 262 and 523 Hz. Each note was presented for 100 ms with 20 ms between notes (Figure 1A). As in the visual distractor task, there were four auditory perceptual load conditions: no distractors presented alongside McGurk stimuli (DF); distractor stimuli were present but not attended (NL), participants were asked to detect a tone significantly higher pitch (1,046–2,093 Hz) than the standard tones (LL); participants were asked to detect notes that were twice the duration of the standard tones (HL). For LL and HL trials, there was a 50% probability that the target would be present in the RSAP stream. After each presentation, participants first responded to the McGurk task, then selected “Yes” or “No” to indicate if they observed the target. Participants completed all perceptual load blocks in one session.

### 2.6. Data analysis

#### 2.6.1. Psychophysical analyses

Responses for incongruent illusory trials on the McGurk task were divided into “visual” (“ga”), “auditory” (“ba”), and “fused” (“da” or “tha”). Percent fused reports were calculated for each participant for each perceptual load condition and distractor modality. We conducted a repeated-measures analysis of variance (RMANOVA) on percent fused reports with load (NL or HL) as a within-subject factor and distractor task modality (visual or auditory) as a between-subjects factor to determine whether increasing perceptual load affected the perception of the McGurk Illusion and whether this effect was modulated by distractor modality.

#### 2.6.2. Sensory noise calculations

Previous models have been developed to determine sensory noise (Magnotti and Beauchamp, 2015, 2017). However, these models do not account for visual and auditory noise independently. Including visual and auditory noise independently permits investigations into how distractors impact precision of information available when forming McGurk percepts, which may be important for understanding attentional influences on multisensory integration. We assessed sensory noise in both modalities using variability in responses to unisensory visual and auditory presentations. Previous studies determined that the encoding of auditory and visual cues follow separate Gaussian distributions and that the variance of that distribution reflects sensory noise (Ma et al., 2009; Magnotti and Beauchamp, 2017). Responses to visual and auditory-only trials were used to estimate sensory noise separately for each experimental condition: syllable presented (“ba,” “tha,” “da,” “ga”), distractor modality (auditory or visual), and perceptual load (DF, NL, or HL). Each response was assigned a value reflecting the reported syllable’s relative location in audiovisual perceptual space (Figure 1B; Ma et al., 2009; Olasagasti et al., 2015; Magnotti and Beauchamp, 2017; Lalonde and Werner, 2019). In line with previous work, fused reports were placed in the middle of “ba” and “ga” (Magnotti and Beauchamp, 2017). However, our study design permitted two options “da” and “tha” for fused responses. To account for differences in between the two syllables we adapted a 10-point scale. This would permit us to separate “tha” and “da,” to accommodate previous findings that “tha” is more similar to “ba,” while “da” is more similar to “ga” (Lalonde and Werner, 2019). Further, Lalonde and Werner identified multiple consonant-groups separating each syllable, thus a 10-point scale would reflect distance in audiovisual space between each syllable.

We bootstrapped 10,000 samples for each participant’s response to each syllable presented during auditory- and visual-only trials (Figure 1C, Stein et al., 2009). We averaged each syllable’s overall visual (σ_*Vis*_) and auditory (σ_*Aud*_) noise for each condition by taking the average sensory noise for all syllables presented during visual or auditory-only trials. Finally, we calculated combined sensory noise to account for both visual and auditory noise. We used the equation: $\sigma_{Combined}=\frac{\sigma_{Vis}-\sigma_{Aud}}{\sigma_{Vis}+\sigma_{Aud}}$, which is based on calculations from maximum likelihood estimate models (Ernst and Banks, 2002) and comparable to models using auditory/visual noise ratio (Magnotti and Beauchamp, 2017; Magnotti et al., 2018). This produces a distribution of combined sensory noise values between 1 and −1, with values >0 indicating that visual noise is greater.

#### 2.6.3. Multiple regression modeling

We developed two multiple linear regression models to determine the effect of sensory noise on McGurk perceptions. We chose to use linear regression because to investigate the roles of attention and sensory noise on the likelihood of perceiving the McGurk effect. Additionally, relevant factors used in the analyses showed significant linear relationships with our dependent factors. The first model investigated factors contributing to McGurk responses at baseline, and the second investigated changes in McGurk responses with increasing perceptual load. All testing and model assessments were carried out in SPSS. First, preliminary model fitting was conducted on data from individuals excluded (*n* = 57) due to poor distractor task performance and lack of unisensory data to explore the relationship between baseline McGurk values and multiple possible predictor variables. These variables included visual noise, auditory noise, distractor modality, accuracy on auditory and visual distractor tasks, and interaction terms. Preliminary results suggested that visual noise, auditory noise, and the combination of the two could be predictive of McGurk responses. After determining potential predictors from excluded data, we then determined whether McGurk responses at baseline (distractor-free condition) correlated with each sensory noise measure (visual, auditory, and combined) to construct the final multiple regression model. Importantly, this baseline regression model allowed us to better contextualize our results and our novel method of estimating sensory noise within modality in the context of previous studies which also relate sensory noise to measures of multisensory integration.

Our second multiple regression analysis modeled the change in McGurk perception from NL to HL (ΔMcGurk = HL McGurk reports − NL McGurk reports). To determine which predictive variables to include, we performed an RMANOVA with visual noise, auditory noise, and combined noise as dependent variables with load (NL and HL) as a within-subjects factor and distractor modality as a between-subjects variable. The variables that were significantly predicted by load were included in a single-step multiple regression model of ΔMcGurk: distractor modality, change in visual noise, and baseline McGurk values. Notably, changes in auditory noise and combined noise were excluded because neither these variables nor their interaction with distractor modality were significantly predicted by load nor did they correlate with changes in McGurk reports across load.

## 3. Results

Participants completed a McGurk detection task to assess their integration of speech stimuli. This task was completed alone (DF) or in addition to a secondary distractor task at various perceptual loads (NL and HL). Participants were separated by which distractor modality (auditory or visual) was presented during the dual-task conditions.

### 3.1. Attentional alterations to McGurk perception

**FIGURE 2:**
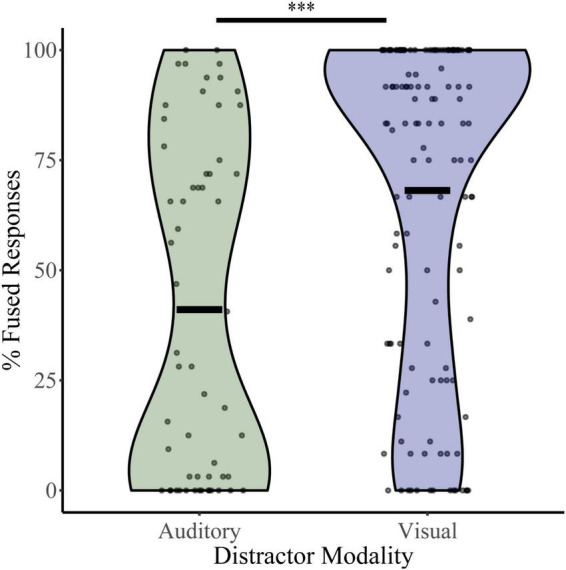
McGurk fused reports for distractor free blocks. The percent of fused reports (“da” or “tha”) during distractor free blocks is shown for each participant for the visual distractor and auditory distractor groups. Horizontal black bars indicate group averages, and violin plots display the distribution of percent fused reports for each task. ^***^Indicates *p* < 0.001.

To assess baseline levels of multisensory integration, percent fused responses (“da” or “tha”) were calculated for illusory trials (auditory “ba” and visual “ga”) during the distractor-free block (Figure 2). Independent *t*-tests revealed significant differences in mean baseline illusory percepts between the auditory distractor group (percent fused = 41.05) and visual distractor group (percent fused = 68.11; *t*_105_ = 4.54, *p* = 1.50 × 10^–5^, Cohen’s *d* = 0.724). These differences were confirmed with bootstrapped (95% CI: 4.45–32.04, *p* = 0.015), non-parametric (U_*N,Aud Dist:* 134; *N, Vis Dist:* 58_ = 2,191, *z* = −4.85, *p* = 1.26 × 10^–6^) and Bayesian (*t*_190_ = 4.81, *p* = 7.4 × 10^–6^, *BF* = 0.00) sample comparisons. Because the distractor-free block was identical for the visual and auditory distractor studies and was most often completed after a NL, LL, or HL block, these results may indicate that McGurk perception is affected by the modality of distractors within the context of the entire task.

**FIGURE 3:**
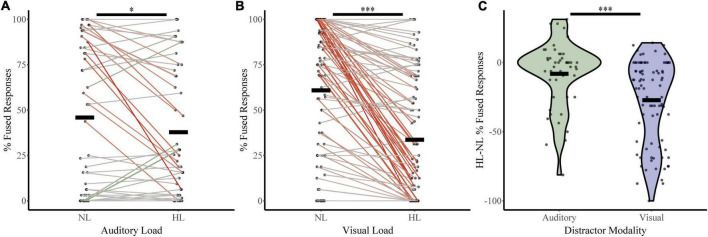
McGurk fused reports for no load (NL) and high load (HL) bocks. The percent of fused reports (“da” or “tha”) during NL and HL blocks are shown for each participant for the auditory distractor **(A)** and visual distractor **(B)** tasks. Horizontal black bars indicate group averages. Colored lines connect individual percent fused reports across each block with a green line indicating an increase in fused reports from NL to HL and a red line indicating a decrease. The difference in percent fused reports across load for rapid serial visual presentation (RSVP) and rapid serial auditory presentation (RSAP) tasks is shown in panel **(C)**. ^***^Indicates *p* < 0.001 and *indicates *p* < 0.05.

To assess how McGurk perception changes with increasing perceptual load, we calculated fused responses during no-load and high load blocks (Figure 3) for both the auditory distractor group (NL %fused = 45.90, HL %fused = 37.80) and visual distractor group (NL %fused = 60.86, HL %fused = 33.68%). A two-way RMANOVA with fused responses as the dependent factor, perceptual load as a within-subjects factor, and distractor modality as a between-subjects factor revealed a main effect of perceptual load (*F*_1,133_ = 48.36, *p* = 1.45 × 10^–10^, partial η^2^ = 0.267) and an interaction between load and distractor modality (*F*_1,133_ = 14.15, *p* = 2.52 × 10^–4^, partial η^2^ = 0.096). We confirmed these findings with *post hoc* two-sample comparisons. These indicate significant changes in McGurk responses from No Load to High Load with visual distractors (*t*_86_ = 8.36, *p* = 9.75 × 10^–13^, Cohen’s d: 0.90; Bootstrapped 95% CI: 20.76–33.72, *p* = 2.00 × 10^–4^; *W* = 114.5, *z* = −6.67, *p* = 2.59 × 10^–11^; *BF* = 0.00). Parametric assessments illustrated a significant change in McGurk responses between auditory No Load to High Load (*t*_47_ = 2.35, *p* = 0.02, Cohen’s d: 0.34; Bootstrapped 95% CI: 1.62–15.09, *p* = 0.032; *BF* = 0.67); however, this effect only approached significance when using non-parametric Wilcoxon comparisons (*W* = 271.50, *z* = −1.86, *p* = 0.06). Further, differences in McGurk reports from No Load to High Load conditions were dependent on distractor modality (*t*_117_ = −4.03, *p* = 1.01 × 10^–4^, Cohen’s *d* = −0.68; Bootstrapped 95% CI: −28.35 to −9.86, *p* = 2.00 × 10^–4^; U_*N,Aud Dist:* 48; *N, Vis Dist:* 87_ = 2,918, *z* = 3.83, *p* = 1.30 × 10^–4^; Bayesian *t*_133_ = −3.8, *p* = 2.532 × 10^–4^, *BF* = 0.012). These results indicate that increasing perceptual load leads to a decrease in integration; however, visual distractors led to a greater decrease in integration than auditory distractors. Supplementary material include figures and statistics for participant distractor task accuracy (Supplementary Figure 1), unisensory and multisensory congruent trial-type accuracy (Supplementary Figure 2), and changes in McGurk reports across NL, LL, and HL (Supplementary Figure 3) for both distractor modalities.

### 3.2. Sensory noise

#### 3.2.1. Baseline sensory noise

**FIGURE 4:**
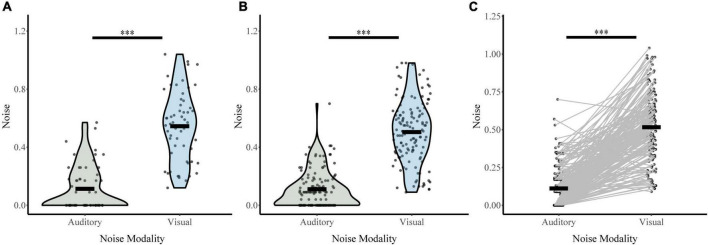
Sensory noise for distractor free blocks. Auditory and visual sensory noise is shown separately for the auditory distractor **(A)** and visual distractor **(B)** groups. Horizontal black bars indicate group averages, and violin plots display the distribution of sensory noise in each modality for each task. Panel **(C)** shows auditory and visual sensory noise for all participants connected for each participant with straight lines. ^***^Indicates *p* < 0.001.

Responses on unisensory trials were used to determine auditory and visual noise values for each participant during baseline conditions (distractor free block; Figure 4). Both visual distractor group (σ_*Vis*_ 0.50, σ_*Aud*_ 0.11) and auditory distractor group (σ_*Vis*_ 0.54, σ_*Aud*_ 0.11) had lower auditory noise than visual noise. A two-way ANOVA with sensory noise as the dependent variable, noise modality as a within-subjects factor, and distractor modality as a between-subjects factor revealed a main effect of noise modality (*F*_1,190_ = 450, *p* = 4.83 × 10^–52^, partial η^2^ = 0.703). There was no effect of distractor modality (*F*_1,190_ = 1.092, *p* = 0.297, partial η^2^ = 0.006) or interaction between noise and distractor modality (*F*_1,190_ = 0.948, *p* = 0.331, partial η^2^ = 0.005). *Post hoc* sample comparisons using *t*-tests and non-parametric assessments corroborated these findings. There were significant differences between baseline auditory and visual noise for individuals in both auditory-distractor (*t*_47_ = 10.93, *p* = 1.70 × 10^–14^, Cohen’s d: 1.58; Bootstrapped 95% CI: 0.34–0.48, *p* = 2.00 × 10^–4^; *W* = 24, *z* = −6.44, *p* = 1.21 × 10^–10^; *t*_57_ = 12.3, *p* = 0.000, *BF* = 0.00) and visual distractor (*t*_86_ = 13.78, *p* = 1.78 × 10^–23^, Cohen’s d: 1.48; Bootstrapped 95% CI: 1.62–15.09, *p* = 0.03; *W* = 89.00, *z* = −9.85, *p* = 0.000; Bayesian *t*_134_ = 19.2, *p* = 0.000, *BF* = 0.00) groups. These results indicate that auditory noise was significantly lower than visual noise regardless of the distractor modality for the task.

#### 3.2.2. Change in sensory noise

**FIGURE 5:**
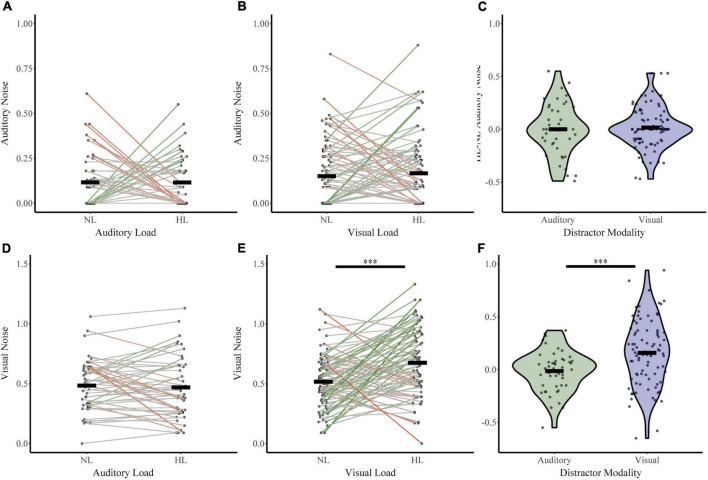
Changes in sensory noise across perceptual load. Auditory noise does not change with increasing auditory **(A)** or visual **(B)** perceptual load. Visual noise increases with increasing visual **(E)** but not auditory **(D)** noise. HL-NL differences in auditory noise **(C)** and visual noise **(F)** confirm that visual load selectively increases visual noise. Horizontal black bars indicate group averages, and violin plots display the distribution of HL-NL sensory noise differences for each distractor and noise modality. ^***^Indicates *p* < 0.001.

Next, we investigated whether perceptual load increased sensory noise and whether this effect was dependent on distractor or noise modality (Figure 5). For the auditory distractor group, auditory noise (NL σ_*Aud*_ 0.12, HL σ_*Aud*_ 0.12) and visual noise (NL σ_*Vis*_ 0.48, HL σ_*Vis*_ 0.47) remained stable across load. For the visual distractor group, auditory noise remained stable (NL σ_*Aud*_ 0.15, HL σ_*Aud*_ 0.17); however, visual noise increased (NL σ_*Vis*_ 0.52, HL σ_*Vis*_ 0.67). An RMANOVA of sensory noise with noise modality and load (NL or HL) as within-subjects factors and distractor modality as a between-subjects factor revealed significant main effects of noise modality (*F*_1,133_ = 414.836, *p* = 1.03 × 10^–42^, partial η^2^ = 0.757), load (*F*_1,133_ = 5.702, *p* = 0.02, partial η^2^ = 0.041), and distractor modality (*F*_1,133_ = 11.816, *p* = 0.001, partial η^2^ = 0.082). There were also significant interactions between load and distractor modality (*F*_1,133_ = 8.06, *p* = 0.005, partial η^2^ = 0.057) and a three-way interaction between noise modality, load, and distractor modality (*F*_1,133_ = 7.612, *p* = 0.007, partial η^2^ = 0.054). The interaction between distractor modality and noise modality approached significance (*F*_1,133_ = 3.890, *p* = 0.051, partial η^2^ = 0.028). *Post-hoc* analyses using *t*-tests and non-parametric assessments corroborated these findings. Visual noise increased from no load to high load in visual modality only (*t*_86_ = −4.78, *p* = 7.28 × 10^–6^, Cohen’s d: −0.51; Bootstrapped 95% CI: −0.22 to −0.09, *p* = 2.00 × 10^–4^; *W* = 2,928, *z* = 4.29, *p* = 1.77 × 10^–5^; *BF* = 0.01). However, visual noise did not significantly change from no load to high load with auditory distractors (*t*_47_ = 0.53, *p* = 0.60, Cohen’s d: 0.08; Bootstrapped 95% CI: −0.04 to 0.07, *p* = 0.60; *W* = 541.5, *z* = −0.238, *p* = 0.81; *BF* = 7.71). As follows, change in visual noise was higher with visual distractors than auditory distractors (*t*_131_ = 4.0, *p* = 1.04 × 10^–4^, Cohen’s *d* = 0.63, Bootstrapped 95% CI: 0.09–0.26; *p* = 2.00 × 10^–4^; U_*N,Aud Dist:* 48; *N, Vis Dist:* 87_ = 1,329, *z* = −3.49, *p* = 4.85 × 10^–4^; Bayesian *t*_133_ = 3.52, *p* = 1.0 × 10^–3^, *BF* = 0.025). Further, auditory noise did not significantly change from no load to high load with either visual (*t*_86_ = −0.74, *p* = 0.46, Cohen’s d: −0.08; Bootstrapped 95% CI: −0.06 to 0.03, *p* = 0.47; *W* = 1,236, *z* = 0.606, *p* = 0.545; *BF* = 9.05) or auditory distractors (*t*_47_ = 0.01, *p* = 1.00, Cohen’s d: 1.0 × 10^–3^; Bootstrapped 95% CI: −0.07 to 0.07, *p* = 1.00; *W* = 331.500, *z* = −0.024, *p* = 0.98; *BF* = 8.86). The difference in auditory noise from no load to high load did not significantly differ between distractor modality (*t*_84_ = 0.40, *p* = 0.69, Cohen’s *d* = 0.08; Bootstrapped 95% CI: −0.06 to 0.09, *p* = 0.70; U_*N,Aud Dist:* 48; *N, Vis Dist:* 87_ = 2,025, *z* = −0.29, *p* = 0.77; *t*_133_ = 0.42, *p* = 0.68, *BF* = 6.17) Collectively, these findings indicate that attentional increases in sensory noise are specific to visual noise with increasing visual load only.

### 3.3. Multiple linear regressions analysis models

#### 3.3.1. Baseline McGurk reports

**FIGURE 6:**
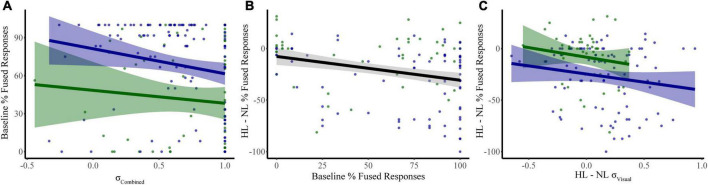
Significant predictors of McGurk fused reports. Our first model identified combined sensory noise and distractor modality as significant predictors of fused reports during baseline conditions (distractor free) **(A)**. Changes in fused reports from NL to HL conditions were related to both baseline McGurk fused reports **(B)** and to the change in visual noise **(C)**. Shaded regions reflect the 95% confidence interval for the regression.

We constructed a multiple linear regression model to determine which sensory noise measures (auditory noise, visual noise, or a combination of both) best predicted baseline McGurk reports. Distractor Modality was included in the model because our RMANOVA analyses (described above) identified it as a significant factor. While neither visual noise (*r*_134_ = 0.028, *p* = 0.701) nor auditory noise (*r*_134_ = 0.118, *p* = 0.104) correlated with baseline McGurk reports, combined noise did significantly correlate with baseline McGurk reports (*r*_134_ = −0.172, *p* = 0.017). Thus, we constructed a multiple regression model to predict baseline McGurk reports with distractor modality and combined noise as factors (Table 1). A significant relationship was found (*F*_2,189_ = 13.24, *p* = 4.16 × 10^–6^) with an R^2^ of 0.123. Baseline McGurk reports were significantly predicted by distractor modality (β = −0.306, *t* = −4.49, *p* = 1.26 × 10^–5^; bootstrap *p* = 0.0002) and combined noise (β = −0.150, *t* = −2.20, *p* = 0.029; bootstrap *p* = 0.049; Figure 6A). Neither auditory noise (Δ*F*_1,188_ = 0.05, *p* = 0.817; ΔR^2^ = 0.0002) nor visual noise (Δ*F*_1,187_ = 3.25, *p* = 0.073; ΔR^2^ = 0.015) significantly increased the predictability of this multiple regression model when added in stepwise fashion, confirming the relative importance of combined noise in predicting baseline McGurk perceptions.

**TABLE 1:** Multiple linear regression: Baseline McGurk reports showing predictive power of distractor modality and combined noise on baseline McGurk perception.

Predictor	Unstandardized coefficients	SE	95% CI (Bootstrapped)		β	*p*-value
			**Lower**	**Upper**		
Intercept	79.16	5.96	66.93	91.42		0.0002
Distractor modality	−26.14	5.83	−37.64	−14.12	−0.306	0.0002
Combined noise	−16.12	7.57	−33.26	−0.40	−0.150	0.049

#### 3.3.2. Dual task McGurk reports

We constructed a multiple linear regression model to determine which factors contributed to changes in McGurk reports with increasing perceptual load. To determine which factors to include, we performed separate RMANOVAs with visual noise, auditory noise, or combined noise as dependent variables, perceptual load as a within-subjects factor, and distractor modality as a between-subjects factor. For visual noise, there was a significant main effect of load (*F*_1,133_ = 8.51, *p* = 0.004, partial η^2^ = 0.060) and distractor modality (*F*_1,133_ = 11.079, *p* = 0.001, partial η^2^ = 0.077) as well as a significant interaction between load and distractor modality (*F*_1,133_ = 12.38, *p* = 0.001, partial η^2^ = 0.085). There were no significant effects for auditory noise (load: *F*_1,133_ = 0.164, *p* = 0.686, partial η^2^ = 0.001; distractor modality: *F*_1,133_ = 3.064, *p* = 0.082, partial η^2^ = 0.023; interaction: *F*_1,133_ = 0.173, *p* = 0.678, partial η^2^ = 0.001) or combined noise (load: *F*_1,133_ = 0.720, *p* = 0.398, partial η^2^ = 0.005; distractor modality: *F*_1,133_ = 0.421, *p* = 0.517, partial η^2^ = 0.003; interaction: *F*_1,133_ = 0.101, *p* = 0.751, partial η^2^ = 0.001). Additionally, the change in McGurk reports from no load to high load significantly correlated with the change in Visual Noise from no load to high load (*r*_134_ = −0.235, *p* = 0.006) and not change in Auditory (*r*_134_ = −0.085, *p* = 0.330) or change in Combined Noise (*r*_134_ = −0.044, *p* = 0.615). Collectively, these results suggest that changes in visual noise across load best explain changes in McGurk perception with increasing load as compared to other measures of sensory noise. Thus, we constructed a multiple linear regression model with change in McGurk reports from no load to high load as the dependent variable and the following potential explanatory variables: baseline McGurk reports, change in visual noise, and distractor modality (Table 2). A significant relationship was found (*F*_3,131_ = 10.32, *p* = 3.81 × 10^–6^) with an R^2^ of 0.191. Change in McGurk reports was significantly predicted by baseline McGurk reports (β = −0.276, *t* = −3.42, *p* = 0.001; bootstrap *p* = 4.00 × 10^–4^; Figure 6B), Distractor Modality (β = 0.197, *t* = 2.33, *p* = 0.021; bootstrap *p* = 0.008), change in Visual Noise (β = −0.184, *t* = −2.24, *p* = 0.027; bootstrap *p* = 0.022; Figure 6C). Neither change in auditory noise (Δ*F*_1,130_ = 0.20, *p* = 0.654; ΔR^2^ = 0.001) nor change in combined noise (Δ*F*_1,129_ = 0.18, *p* = 0.672; ΔR^2^ = 0.001) increased the predictability of this multiple regression model when added in stepwise fashion, confirming the relative importance of changes in visual noise predicting atentional disruptions to McGurk perceptions.

**TABLE 2:** Multiple linear regression: Dual-task McGurk reports showing predictive power of distractor modality, baseline McGurk, and Δvisual noise on ΔMcGurk responses from NL to HL.

Predictor	Unstandardized coefficients	SE	95% CI (Bootstrap)		β	*p*-value (Bootstrap)
			**Lower**	**Upper**		
Intercept	−11.61	4.95	−19.31	−5.07		0.001
Baseline McGurk	−0.20	0.06	−0.30	−0.10	−0.276	0.0004
Distractor modality	12.10	5.20	3.00	20.91	0.197	0.008
ΔVisual noise	−19.13	8.60	−36.43	−3.90	−0.184	0.022

## 4. Discussion

The present study investigated whether variations in sensory noise could explain the impact of attention on multisensory integration of speech stimuli and to what extent this mechanism operates in a modality-specific manner. To examine within-modality effects, we created a novel method of measuring sensory noise based on response variability in unisensory trials. Importantly, this method expands on previous models, allowing us to investigate the effects of visual and auditory noise independently from one another. Consistent with other computational models of multisensory speech integration, the overwhelming majority of participants had higher visual noise compared to auditory (Massaro, 1999; Ma et al., 2009; Magnotti and Beauchamp, 2015, 2017; Magnotti et al., 2020). Additionally, our combined sensory noise measure, which is the direct equivalent of the sensory noise ratio in the CIMS model (Magnotti and Beauchamp, 2017; Magnotti et al., 2020), was a better predictor of baseline McGurk reports than visual or auditory noise alone. These findings are strongly aligned with other computational measures of sensory noise and lend evidence to the overall importance of sensory noise for multisensory integration. The novel method of estimating sensory noise separately for each modality provides additional functionality to current models of multisensory speech integration which primarily rely on the relative levels of visual and auditory noise but do not permit either to vary independently (Magnotti and Beauchamp, 2017). These within-modality measures of sensory noise allowed us to identify that changes in visual noise, specifically, were associated with attentional modulations to multisensory speech perception. Increases in visual load led to increased visual noise and decreased McGurk perception. Correspondingly, changes in visual noise were predictive of changes to McGurk reports across load. These findings suggest that attention alters the encoding of visual speech information and that attention may impact sensory noise in a modality-specific manner. Unfortunately, our method of calculating sensory noise resulted in many participants having an auditory noise value of zero even under high perceptual load, suggesting that this method may not be sensitive enough to estimate very low levels of sensory noise. However, it can accurately determine the individual contributions of and changes to auditory and visual noise on multisensory integration.

Our results strongly indicate that modulations of attention differentially impact multisensory speech perception depending on the sensory modality of the attentional manipulation. While we found striking increases in visual noise with increasing visual load, we did not find corresponding increases in auditory noise with increasing auditory load suggesting a separate mechanism by which auditory attention influences multisensory speech integration. Additionally, while increasing perceptual load led to decreased McGurk reports for both visual and auditory secondary tasks, this effect was more pronounced for the visual task suggesting that alterations to visual attention may have a heightened impact on multisensory speech integration. Because the auditory and visual secondary tasks differed in ways other than their modality, we cannot eliminate the possibility that these differences account for our observed modality effects. We hypothesize that our visual secondary task engages featural attention, and although our secondary auditory task asked participants to identify auditory features (i.e., pitch and duration), we suspect that participants listened for melodic or rhythmic indicators of targets which may have engaged object-based attention. Future research is needed to investigate the relative contributions of distractor modality and type of attentional manipulation on multisensory speech integration. Another potential explanation for distractor modality effects is differential patterns of eye movements. Gaze behavior has been shown to influence the McGurk effect (Paré et al., 2003; Gurler et al., 2015; Jensen et al., 2018; Wahn et al., 2021). Because eye movements were not monitored during this study, future research is needed to investigate whether gaze behavior may explain modality differences in the impact of the secondary task on multisensory speech integration. Surprisingly, McGurk reports differed in the distractor-free condition across auditory vs. visual secondary task groups even though the tasks were identical. This suggests that the sensory modality of a secondary task may influence multisensory speech perception even when not concurrently presented. Approximately 70% of participants completed the distractor-free block after a low load or high load block, suggesting that our secondary task may prime attention to its corresponding modality and subsequently alter speech integration. Interestingly, we did not find differences in sensory noise across distractor modality in the distractor-free condition. This implies that any task context effects may lead to changes in participants’ priors or relative weighing of auditory vs. visual speech information (Shams et al., 2005; Kayser and Shams, 2015; Magnotti and Beauchamp, 2017; Magnotti et al., 2020). The current study was not designed to assess order effects; thus, future research is needed to fully investigate modality-specific priming effects and to elucidate the mechanisms by which they may influence multisensory speech perception.

The results of this study inform our understanding of the mechanisms by which attention influences multisensory processing. Multisensory speech integration relies on both extensive processing of the auditory and visual speech signal and convergence of auditory and visual pathways onto multisensory cortical sites such as the Superior Temporal Sulcus (STS) (Beauchamp et al., 2004, 2010; Callan et al., 2004; Nath and Beauchamp, 2011, 2012; O’Sullivan et al., 2019, 2021; Ahmed et al., 2021; Nidiffer et al., 2021). Additionally, the functional connectivity between STS and unisensory cortices differs according to the reliability of the corresponding unisensory information (e.g., increased visual reliability will lead to increased functional connectivity between visual cortex and STS) (Nath and Beauchamp, 2011). Our findings suggest that increasing visual load leads to disrupted encoding of the visual speech signal which then leads to a deweighting of visual information potentially through decreased functional connectivity between the STS and visual cortex. Interestingly, increasing auditory load does not appear to disrupt multisensory speech integration through the same mechanism. Ahmed et al. found that attention favors integration at later stages of speech processing (Ahmed et al., 2021) suggesting that our secondary auditory task may disrupt later stages of integrative processing. Future research utilizing neuroimaging methodology is needed to link behavioral estimates of sensory noise to specific neural mechanisms.

Identifying the specific neural mechanisms by which top-down cognitive factors shape multisensory processing is important for our understanding of how multisensory integration functions in realistic contexts and across individual differences. For example, older adults exhibit either intact, enhanced, or shifted patterns of multisensory integration depending on the task utilized in the study (Hugenschmidt et al., 2009; Freiherr et al., 2013; de Dieuleveult et al., 2017; Parker and Robinson, 2018). Interestingly, several studies have shown altered sensory dominance and weighting of unisensory information in older adults when compared to younger adults (Murray et al., 2018; Jones and Noppeney, 2021). Within-modality measures of sensory noise as described in this study may help to illuminate the reasons why certain multisensory stimuli and tasks lead to differences in the multisensory effects observed in the aging population. Cognitive control mechanisms are also known to decline with healthy aging, and manipulations of attention (e.g., dual-task designs) consistently have a larger impact on the elderly (Mahoney et al., 2012; Carr et al., 2019; Ward et al., 2021). Currently, there is a gap in knowledge on how attention may alter relative sensory weighting in older adults that could be addressed by utilizing the experimental design described in this study. Addressing this gap in knowledge could improve our understanding of multisensory speech integration in normal aging and with sensory loss (Peter et al., 2019; Dias et al., 2021) as well as current multisensory screening tools for assessing risks for falls in the elderly (Mahoney et al., 2019; Zhang et al., 2020). In addition to healthy aging, many developmental disorders are characterized by disruptions to both multisensory functioning and attention, and these neurological processes may interact to worsen the severity of these disorders (Belmonte and Yurgelun-Todd, 2003; de Jong et al., 2010; Kwakye et al., 2011; Magnée et al., 2011; Harrar et al., 2014; Krause, 2015; Mayer et al., 2015; Noel et al., 2018b). Previous research indicates that deficits in processing both speech (van Laarhoven et al., 2019) and non-speech (Leekam et al., 2007) stimuli were present in subjects on the autism spectrum. Sensory noise and its interactions with attention may contribute to differences in ASD sensory processing beyond stimulus signal-to-noise ratio or general neural noise. Investigating these mechanisms may help us understand and identify disruptions in the relationship between multisensory integration and attention, inspiring new strategies for interventions to address altered functioning in these disorders.

## Data availability statement

The raw data supporting the conclusions of this article will be made available by the authors, without undue reservation.

## Ethics statement

The studies involving human participants were reviewed and approved by the Oberlin College Institutional Review Board. The patients/participants provided their written informed consent to participate in this study.

## Author contributions

LK, VF, CD, EP, WK, and CN contributed to the conception and design of the project and wrote the manuscript. LK and CD collected the data for the project. LK, VF, and CN developed the sensory noise analysis, and analyzed and interpreted the final data for the manuscript. CD, EP, and WK contributed to initial data analysis and interpretation. All authors contributed to the article and approved the submitted version.

## Supplementary material

Supplementary Figure 1 Performance on low-load (LL) and high-load (HL) distractor tasks. Participants scored significantly higher during the LL conditions than the HL conditions for both auditory (**A**; *LL* = 92.6; *HL* = 78.6; *t*_44_ = 10.39, *p* = 2.04 × 10^–13^, Cohen’s *d* = 1.79) and visual (**B**; *LL* = 94.0; *HL* = 85.4; t_76_ = 13.81, *p* = 2.02 × 10^–22^, Cohen’s *d* = 1.48) distractor tasks.

Supplementary Figure 2 Performance on unisensory and congruent multisensory trials for no load (NL), low load (LL), and high load (HL) blocks. Percent correct syllable identification for visual-only, auditory-only, and multisensory congruent trials for the auditory distractor **(A)** and visual distractor **(B)** tasks. An RMANOVA revealed that distractor modality (*F*_1,128_ = 12.8, *p* = 4.91 × 10^–4^, partial η^2^ = 0.091), perceptual load (*F*_2,256_ = 6.7, *p* = 0.001, partial η^2^ = 0.050), and syllable modality (*F*_2,256_ = 1040.5, *p* = 1.16 × 10^–123^, partial η^2^ = 0.890) significantly altered accuracy.

Supplementary Figure 3 McGurk fused reports for no load (NL), low load (LL), and high load (HL). The percent of fused reports (“da” or “tha”) during each block are shown for auditory distractor **(A)** and visual distractor **(B)** tasks. Horizontal bars indicate group averages. Colored lines connect individual percent fused reports across each block. Green lines indicate increased in fused reports and a red line indicates a decrease in fused reports. An RMANOVA revealed that both perceptual load (*F*_2,256_ = 22.5, *p* = 9.90 × 10^–10^, partial η^2^ = 0.148) and the interaction between load and distractor modality (*F*_2,256_ = 4.7, *p* = 0.010, partial η^2^ = 0.035) significantly altered percent McGurk reports.

## References

[B1] Ahmed F. Nidiffer A. R. O’Sullivan A. E. Zuk N. J. Lalor E. C. (2021). The integration of continuous audio and visual speech in a cocktail-party environment depends on attention. *bioRxiv* [Preprint] 10.1101/2021.02.10.430634 PMC12956257 37121375

[B2] Al-Aidroos N. Said C. P. Turk-Browne N. B. (2012). Top-down attention switches coupling between low-level and high-level areas of human visual cortex. *Proc. Natl. Acad. Sci. U.S.A.* 109 14675–14680. 10.1073/pnas.1202095109 22908274 PMC3437858

[B3] Alsius A. Möttönen R. Sams M. E. Soto-Faraco S. Tiippana K. (2014). Effect of attentional load on audiovisual speech perception: Evidence from ERPs. *Front. Psychol.* 5:727. 10.3389/fpsyg.2014.00727 25076922 PMC4097954

[B4] Alsius A. Navarra J. Campbell R. Soto-Faraco S. (2005). Audiovisual integration of speech falters under high attention demands. *Curr. Biol.* 15 839–843. 10.1016/j.cub.2005.03.046 15886102

[B5] Alsius A. Navarra J. Soto-Faraco S. (2007). Attention to touch weakens audiovisual speech integration. *Exp. Brain Res.* 183 399–404. 10.1007/s00221-007-1110-1 17899043

[B6] Beauchamp M. S. Argall B. D. Bodurka J. Duyn J. H. Martin A. (2004). Unraveling multisensory integration: Patchy organization within human STS multisensory cortex. *Nat. Neurosci.* 7 1190–1192. 10.1038/nn1333 15475952

[B7] Beauchamp M. S. Nath A. R. Pasalar S. (2010). FMRI-Guided transcranial magnetic stimulation reveals that the superior temporal sulcus is a cortical locus of the McGurk effect. *J. Neurosci.* 30 2414–2417. 10.1523/JNEUROSCI.4865-09.2010 20164324 PMC2844713

[B8] Belmonte M. K. Yurgelun-Todd D. A. (2003). Functional anatomy of impaired selective attention and compensatory processing in autism. *Brain Res. Cogn. Brain Res.* 17 651–664. 10.1016/S0926-6410(03)00189-7 14561452

[B9] Bobrowski O. Meir R. Eldar Y. C. (2009). Bayesian filtering in spiking neural networks: Noise, adaptation, and multisensory integration. *Neural. Comput.* 21 1277–1320. 10.1162/neco.2008.01-08-692 19018706

[B10] Bonato M. Spironelli C. Lisi M. Priftis K. Zorzi M. (2015). Effects of multimodal load on spatial monitoring as revealed by ERPs. *PLoS One* 10:e0136719. 10.1371/journal.pone.0136719 26335779 PMC4559441

[B11] Burns J. K. Blohm G. (2010). Multi-sensory weights depend on contextual noise in reference frame transformations. *Front. Hum. Neurosci.* 4:221. 10.3389/fnhum.2010.00221 21165177 PMC3002464

[B12] Callan D. E. Jones J. A. Munhall K. Kroos C. Callan A. M. Vatikiotis-Bateson E. (2004). Multisensory integration sites identified by perception of spatial wavelet filtered visual speech gesture information. *J. Cogn. Neurosci.* 16 805–816. 10.1162/089892904970771 15200708

[B13] Carr S. Pichora-Fuller M. K. Li K. Z. H. Phillips N. Campos J. L. (2019). Multisensory, multi-tasking performance of older adults with and without subjective cognitive decline. *Multisens. Res.* 32 797–829. 10.1163/22134808-20191426 31277054

[B14] Cascio C. N. O’Donnell M. B. Tinney F. J. Lieberman M. D. Taylor S. E. Strecher V. J. (2016). Self-affirmation activates brain systems associated with self-related processing and reward and is reinforced by future orientation. *Soc. Cogn. Affect. Neurosci.* 11 621–629. 10.1093/scan/nsv136 26541373 PMC4814782

[B15] Dean C. L. Eggleston B. A. Gibney K. D. Aligbe E. Blackwell M. Kwakye L. D. (2017). Auditory and visual distractors disrupt multisensory temporal acuity in the crossmodal temporal order judgment task. *PLoS One* 12:e0179564. 10.1371/journal.pone.0179564 28723907 PMC5516972

[B16] Deneve S. Pouget A. (2004). Bayesian multisensory integration and cross-modal spatial links. *J. Physiol. Paris* 98 249–258. 10.1016/j.jphysparis.2004.03.011 15477036

[B17] Dias J. W. McClaskey C. M. Harris K. C. (2021). Audiovisual speech is more than the sum of its parts: Auditory-visual superadditivity compensates for age-related declines in audible and lipread speech intelligibility. *Psychol. Aging* 36 520–530. 10.1037/pag0000613 34124922 PMC8427734

[B18] de Dieuleveult A. L. Siemonsma P. C. van Erp J. B. F. Brouwer A.-M. (2017). Effects of aging in multisensory integration: A systematic review. *Front. Aging Neurosci.* 9:80. 10.3389/fnagi.2017.00080 28400727 PMC5368230

[B19] Driver J. (2001). A selective review of selective attention research from the past century. *Br. J. Psychol.* 1 53–78. 10.1348/000712601162103 11802865

[B20] de Jong J. J. Hodiamont P. P. G. de Gelder B. (2010). Modality-specific attention and multisensory integration of emotions in schizophrenia: Reduced regulatory effects. *Schizophr. Res.* 122 136–143. 10.1016/j.schres.2010.04.010 20554159

[B21] Engel A. K. Senkowski D. Schneider T. R. (2012). “Multisensory integration through neural coherence,” in *The Neural Bases of Multisensory Processes* Frontiers in Neuroscience, eds Murray M. M. Wallace M. T. Murray M. M. Wallace M. T. Murray M. M. Wallace M. T. (Boca Raton, FL: CRC Press). 22593880

[B22] Ernst M. O. Banks M. S. (2002). Humans integrate visual and haptic information in a statistically optimal fashion. *Nature* 415 429–433. 10.1038/415429a 11807554

[B23] Freiherr J. Lundström J. N. Habel U. Reetz K. (2013). Multisensory integration mechanisms during aging. *Front. Hum. Neurosci.* 7:863. 10.3389/fnhum.2013.00863 24379773 PMC3861780

[B24] Friese U. Daume J. Göschl F. König P. Wang P. Engel A. K. (2016). Oscillatory brain activity during multisensory attention reflects activation, disinhibition, and cognitive control. *Sci. Rep.* 6:32775. 10.1038/srep32775 27604647 PMC5015072

[B25] Gibney K. D. Aligbe E. Eggleston B. A. Nunes S. R. Kerkhoff W. G. Dean C. L. (2017). Visual distractors disrupt audiovisual integration regardless of stimulus complexity. *Front. Integr. Neurosci.* 11:1. 10.3389/fnint.2017.00001 28163675 PMC5247431

[B26] Gomez-Ramirez M. Kelly S. P. Molholm S. Sehatpour P. Schwartz T. H. Foxe J. J. (2011). Oscillatory sensory selection mechanisms during intersensory attention to rhythmic auditory and visual inputs: A human electrocorticographic investigation. *J. Neurosci.* 31 18556–18567. 10.1523/JNEUROSCI.2164-11.2011 22171054 PMC3298747

[B27] Gurler D. Doyle N. Walker E. Magnotti J. Beauchamp M. (2015). A link between individual differences in multisensory speech perception and eye movements. *Atten. Percept. Psychophys.* 77 1333–1341. 10.3758/s13414-014-0821-1 25810157 PMC4437244

[B28] Harrar V. Tammam J. Pérez-Bellido A. Pitt A. Stein J. Spence C. (2014). Multisensory integration and attention in developmental dyslexia. *Curr. Biol.* 24 531–535. 10.1016/j.cub.2014.01.029 24530067

[B29] Hugenschmidt C. E. Mozolic J. L. Laurienti P. J. (2009). Suppression of multisensory integration by modality-specific attention in aging. *Neuroreport* 20 349–353. 10.1097/WNR.0b013e328323ab07 19218871 PMC2692738

[B30] Jensen A. Merz S. Spence C. Frings C. (2018). Overt spatial attention modulates multisensory selection. *J. Exp. Psychol. Hum. Percept. Perform.* 45 174–188. 10.1037/xhp0000595 30589358

[B31] Jones S. A. Noppeney U. (2021). Ageing and multisensory integration: A review of the evidence, and a computational perspective. *Cortex* 138 1–23. 10.1016/j.cortex.2021.02.001 33676086

[B32] Kayser C. Shams L. (2015). Multisensory causal inference in the brain. *PLoS Biol.* 13:e1002075. 10.1371/journal.pbio.1002075 25710476 PMC4339834

[B33] Keil J. Pomper U. Senkowski D. (2016). Distinct patterns of local oscillatory activity and functional connectivity underlie intersensory attention and temporal prediction. *Cortex* 74 277–288. 10.1016/j.cortex.2015.10.023 26716405

[B34] Koelewijn T. Bronkhorst A. Theeuwes J. (2010). Attention and the multiple stages of multisensory integration: A review of audiovisual studies. *Acta Psychol.* 134 372–384. 10.1016/j.actpsy.2010.03.010 20427031

[B35] Körding K. P. Beierholm U. Ma W. J. Quartz S. Tenenbaum J. B. Shams L. (2007). Causal inference in multisensory perception. *PLoS One* 2:e943. 10.1371/journal.pone.0000943 17895984 PMC1978520

[B36] Krause M. B. (2015). Pay Attention!: Sluggish multisensory attentional shifting as a core deficit in developmental dyslexia. *Dyslexia* 21 285–303. 10.1002/dys.1505 26338085

[B37] Kwakye L. D. Foss-Feig J. H. Cascio C. J. Stone W. L. Wallace M. T. (2011). Altered auditory and multisensory temporal processing in autism spectrum disorders. *Front. Integr. Neurosci.* 4:129. 10.3389/fnint.2010.00129 21258617 PMC3024004

[B38] Lakatos P. Karmos G. Mehta A. D. Ulbert I. Schroeder C. E. (2008). Entrainment of neuronal oscillations as a mechanism of attentional selection. *Science* 320 110–113. 10.1126/science.1154735 18388295

[B39] Lalonde K. Werner L. A. (2019). Perception of incongruent audiovisual English consonants. *PLoS One* 14:e0213588. 10.1371/journal.pone.0213588 30897109 PMC6428273

[B40] Lavie N. Ro T. Russell C. (2003). The role of perceptual load in processing distractor faces. *Psychol. Sci.* 14 510–515. 10.1111/1467-9280.03453 12930485

[B41] Leekam S. R. Nieto C. Libby S. J. Wing L. Gould J. (2007). Describing the sensory abnormalities of children and adults with autism. *J. Autism Dev. Disord.* 37 894–910. 10.1007/s10803-006-0218-7 17016677

[B42] Ling S. Liu T. Carrasco M. (2009). How spatial and feature-based attention affect the gain and tuning of population responses. *Vision Res.* 49 1194–1204. 10.1016/j.visres.2008.05.025 18590754 PMC2696585

[B43] Magnée M. J. C. M. de Gelder B. van Engeland H. Kemner C. (2011). Multisensory integration and attention in autism spectrum disorder: Evidence from event-related potentials. *PLoS One* 6:e24196. 10.1371/journal.pone.0024196 21887382 PMC3161097

[B44] Magnotti J. F. Beauchamp M. S. (2017). A causal inference model explains perception of the mcgurk effect and other incongruent audiovisual speech. *PLoS Comput. Biol.* 13:e1005229. 10.1371/journal.pcbi.1005229 28207734 PMC5312805

[B45] Magnotti J. F. Beauchamp M. S. (2015). The noisy encoding of disparity model of the McGurk effect. *Psychon. Bull. Rev.* 22 701–709. 10.3758/s13423-014-0722-2 25245268 PMC4370809

[B46] Magnotti J. F. Dzeda K. B. Wegner-Clemens K. Rennig J. Beauchamp M. S. (2020). Weak observer-level correlation and strong stimulus-level correlation between the McGurk effect and audiovisual speech-in-noise: A causal inference explanation. *Cortex* 133 371–383. 10.1016/j.cortex.2020.10.002 33221701 PMC8592674

[B47] Magnotti J. F. Ma W. J. Beauchamp M. S. (2013). Causal inference of asynchronous audiovisual speech. *Front. Psychol.* 4:798. 10.3389/fpsyg.2013.00798 24294207 PMC3826594

[B48] Magnotti J. F. Smith K. B. Salinas M. Mays J. Zhu L. L. Beauchamp M. S. (2018). A causal inference explanation for enhancement of multisensory integration by co-articulation. *Sci. Rep.* 8:18032. 10.1038/s41598-018-36772-8 30575791 PMC6303389

[B49] Mahoney J. R. Cotton K. Verghese J. (2019). Multisensory integration predicts balance and falls in older adults. *J. Gerontol. A Biol. Sci. Med. Sci.* 74 1429–1435. 10.1093/gerona/gly245 30357320 PMC6696711

[B50] Mahoney J. R. Verghese J. Dumas K. Wang C. Holtzer R. (2012). The effect of multisensory cues on attention in aging. *Brain Res.* 1472 63–73. 10.1016/j.brainres.2012.07.014 22820295 PMC3592377

[B51] Mangun G. R. (1995). Neural mechanisms of visual selective attention. *Psychophysiology* 32 4–18. 10.1111/j.1469-8986.1995.tb03400.x 7878167

[B52] Massaro D. W. (1999). Speechreading: Illusion or window into pattern recognition. *Trends Cogn. Sci.* 3 310–317. 10.1016/S1364-6613(99)01360-1 10431185

[B53] Mayer A. R. Hanlon F. M. Teshiba T. M. Klimaj S. D. Ling J. M. Dodd A. B. (2015). An fMRI study of multimodal selective attention in schizophrenia. *Br. J. Psychiatry* 207 420–428. 10.1192/bjp.bp.114.155499 26382953 PMC4629072

[B54] Ma W. J. Zhou X. Ross L. A. Foxe J. J. Parra L. C. (2009). Lip-reading aids word recognition most in moderate noise: A bayesian explanation using high-dimensional feature space. *PLoS One* 4:e4638. 10.1371/journal.pone.0004638 19259259 PMC2645675

[B55] Mcgurk H. Macdonald J. (1976). Hearing lips and seeing voices. *Nature* 264 746–748. 10.1038/264746a0 1012311

[B56] Mishra J. Gazzaley A. (2012). Attention distributed across sensory modalities enhances perceptual performance. *J. Neurosci.* 32 12294–12302. 10.1523/JNEUROSCI.0867-12.2012 22933811 PMC3449148

[B57] Mishra J. Martínez A. Hillyard S. A. (2010). Effect of attention on early cortical processes associated with the sound-induced extra flash illusion. *J. Cogn. Neurosci.* 22 1714–1729. 10.1162/jocn.2009.21295 19583464

[B58] Mitchell J. F. Sundberg K. A. Reynolds J. H. (2007). Differential attention-dependent response modulation across cell classes in macaque visual area V4. *Neuron* 55 131–141. 10.1016/j.neuron.2007.06.018 17610822

[B59] Morís Fernández L. Visser M. Ventura-Campos N. Ávila C. Soto-Faraco S. (2015). Top-down attention regulates the neural expression of audiovisual integration. *Neuroimage* 119 272–285. 10.1016/j.neuroimage.2015.06.052 26119022

[B60] Mozolic J. L. Hugenschmidt C. E. Peiffer A. M. Laurienti P. J. (2008). Modality-specific selective attention attenuates multisensory integration. *Exp. Brain Res.* 184 39–52. 10.1007/s00221-007-1080-3 17684735

[B61] Murray M. M. Eardley A. F. Edginton T. Oyekan R. Smyth E. Matusz P. J. (2018). Sensory dominance and multisensory integration as screening tools in aging. *Sci. Rep.* 8:8901. 10.1038/s41598-018-27288-2 29891964 PMC5995929

[B62] Nath A. R. Beauchamp M. S. (2012). A neural basis for interindividual differences in the McGurk effect, a multisensory speech illusion. *Neuroimage* 59 781–787. 10.1016/j.neuroimage.2011.07.024 21787869 PMC3196040

[B63] Nath A. R. Beauchamp M. S. (2011). Dynamic changes in superior temporal sulcus connectivity during perception of noisy audiovisual speech. *J. Neurosci.* 31 1704–1714. 10.1523/JNEUROSCI.4853-10.2011 21289179 PMC3050590

[B64] Nidiffer A. R. Cao C. Z. O’Sullivan A. E. Lalor E. C. (2021). A linguistic representation in the visual system underlies successful lipreading. *bioRxiv* [Preprint] 10.1101/2021.02.09.430299 PMC12956258 37757989

[B65] Noel J.-P. Serino A. Wallace M. T. (2018a). Increased neural strength and reliability to audiovisual stimuli at the boundary of peripersonal space. *J. Cogn. Neurosci.* 31 1155–1172. 10.1162/jocn_a_01334 30188779

[B66] Noel J.-P. Stevenson R. A. Wallace M. T. (2018b). Atypical audiovisual temporal function in autism and schizophrenia: Similar phenotype, different cause. *Eur. J. Neurosci.* 47 1230–1241. 10.1111/ejn.13911 29575155 PMC5980744

[B67] O’Sullivan A. E. Crosse M. J. Liberto G. M. D. de Cheveigné A. Lalor E. C. (2021). Neurophysiological indices of audiovisual speech processing reveal a hierarchy of multisensory integration effects. *J. Neurosci.* 41 4991–5003. 10.1523/JNEUROSCI.0906-20.2021 33824190 PMC8197638

[B68] O’Sullivan A. E. Lim C. Y. Lalor E. C. (2019). Look at me when I’m talking to you: Selective attention at a multisensory cocktail party can be decoded using stimulus reconstruction and alpha power modulations. *Eur. J. Neurosci.* 50 3282–3295. 10.1111/ejn.14425 31013361

[B69] Odegaard B. Wozny D. R. Shams L. (2016). The effects of selective and divided attention on sensory precision and integration. *Neurosci. Lett.* 614 24–28. 10.1016/j.neulet.2015.12.039 26742638

[B70] Okamoto H. Stracke H. Wolters C. H. Schmael F. Pantev C. (2007). Attention improves population-level frequency tuning in human auditory cortex. *J. Neurosci.* 27 10383–10390. 10.1523/JNEUROSCI.2963-07.2007 17898210 PMC6673146

[B71] Olasagasti I. Bouton S. Giraud A.-L. (2015). Prediction across sensory modalities: A neurocomputational model of the McGurk effect. *Cortex* 68 61–75. 10.1016/j.cortex.2015.04.008 26009260

[B72] Paré M. Richler R. C. ten Hove M. Munhall K. G. (2003). Gaze behavior in audiovisual speech perception: The influence of ocular fixations on the McGurk effect. *Percept. Psychophys.* 65 553–567. 10.3758/bf03194582 12812278

[B73] Parker J. L. Robinson C. W. (2018). Changes in multisensory integration across the life span. *Psychol. Aging* 33 545–558. 10.1037/pag0000244 29756807

[B74] Pessoa L. Kastner S. Ungerleider L. G. (2003). Neuroimaging studies of attention: From modulation of sensory processing to top-down control. *J. Neurosci.* 23 3990–3998. 10.1523/JNEUROSCI.23-10-03990.2003 12764083 PMC6741071

[B75] Peter M. G. Porada D. K. Regenbogen C. Olsson M. J. Lundström J. N. (2019). Sensory loss enhances multisensory integration performance. *Cortex* 120 116–130. 10.1016/j.cortex.2019.06.003 31299497

[B76] Schroeder C. E. Lakatos P. Kajikawa Y. Partan S. Puce A. (2008). Neuronal oscillations and visual amplification of speech. *Trends Cogn. Sci.* 12 106–113. 10.1016/j.tics.2008.01.002 18280772 PMC3987824

[B77] Schwartz J.-L. Tiippana K. Andersen T. (2010). *Disentangling Unisensory from Fusion Effects in the Attentional Modulation of McGurk Effects: A Bayesian Modeling Study Suggests That Fusion is Attention-Dependent.* Lyon: HAL.

[B78] Senkowski D. Talsma D. Herrmann C. S. Woldorff M. G. (2005). Multisensory processing and oscillatory gamma responses: Effects of spatial selective attention. *Exp. Brain Res.* 166 411–426. 10.1007/s00221-005-2381-z 16151775

[B79] Shams L. Ma W. J. Beierholm U. (2005). Sound-induced flash illusion as an optimal percept. *Neuroreport* 16 1923–1927. 10.1097/01.wnr.0000187634.68504.bb 16272880

[B80] Soto-Faraco S. Alsius A. (2009). Deconstructing the McGurk-MacDonald illusion. *J. Exp. Psychol. Hum. Percept. Perform.* 35 580–587. 10.1037/a0013483 19331510

[B81] Stein B. E. Stanford T. R. Ramachandran R. Perrault T. J. Rowland B. A. (2009). Challenges in quantifying multisensory integration: Alternative criteria, models, and inverse effectiveness. *Exp. Brain Res.* 198 113–126. 10.1007/s00221-009-1880-8 19551377 PMC3056521

[B82] Stevenson R. A. Baum S. H. Segers M. Ferber S. Barense M. D. Wallace M. T. (2017). Multisensory speech perception in autism spectrum disorder: From phoneme to whole-word perception. *Autism Res.* 10 1280–1290. 10.1002/aur.1776 28339177 PMC5513806

[B83] Stolte M. Bahrami B. Lavie N. (2014). High perceptual load leads to both reduced gain and broader orientation tuning. *J. Vis.* 14:9. 10.1167/14.3.9 PMC3948377 24610952

[B84] Talsma D. Doty T. J. Woldorff M. G. (2007). Selective attention and audiovisual integration: Is attending to both modalities a prerequisite for early integration? *Cereb. Cortex* 17 679–690. 10.1093/cercor/bhk016 16707740

[B85] Talsma D. Senkowski D. Soto-Faraco S. Woldorff M. G. (2010). The multifaceted interplay between attention and multisensory integration. *Trends Cogn. Sci.* 14 400–410. 10.1016/j.tics.2010.06.008 20675182 PMC3306770

[B86] Talsma D. Woldorff M. G. (2005). Selective attention and multisensory integration: Multiple phases of effects on the evoked brain activity. *J. Cogn. Neurosci.* 17 1098–1114. 10.1162/0898929054475172 16102239

[B87] Tang X. Wu J. Shen Y. (2016). The interactions of multisensory integration with endogenous and exogenous attention. *Neurosci. Biobehav. Rev.* 61 208–224. 10.1016/j.neubiorev.2015.11.002 26546734 PMC4753360

[B88] van Laarhoven T. Stekelenburg J. J. Vroomen J. (2019). Increased sub-clinical levels of autistic traits are associated with reduced multisensory integration of audiovisual speech. *Sci. Rep.* 9:9535. 10.1038/s41598-019-46084-0 31267024 PMC6606565

[B89] Wahn B. Schmitz L. Kingstone A. Böckler-Raettig A. (2021). When eyes beat lips: Speaker gaze affects audiovisual integration in the McGurk illusion. *Psychol. Res.* 86 1930–1943. 10.1007/s00426-021-01618-y 34854983 PMC9363401

[B90] Ward N. Menta A. Ulichney V. Raileanu C. Wooten T. Hussey E. K. (2021). The specificity of cognitive-motor dual-task interference on balance in young and older adults. *Front. Aging Neurosci.* 13:804936. 10.3389/fnagi.2021.804936 35087396 PMC8786904

[B91] Woldorff M. G. Gallen C. C. Hampson S. A. Hillyard S. A. Pantev C. Sobel D. (1993). Modulation of early sensory processing in human auditory cortex during auditory selective attention. *Proc. Natl. Acad. Sci. U.S.A.* 90 8722–8726. 10.1073/pnas.90.18.8722 8378354 PMC47430

[B92] Zhang S. Xu W. Zhu Y. Tian E. Kong W. (2020). Impaired multisensory integration predisposes the elderly people to fall: A systematic review. *Front. Neurosci.* 14:411. 10.3389/fnins.2020.00411 32410958 PMC7198912

[B93] Zion Golumbic E. Cogan G. B. Schroeder C. E. Poeppel D. (2013). Visual input enhances selective speech envelope tracking in auditory cortex at a “cocktail party”. *J. Neurosci.* 33 1417–1426. 10.1523/JNEUROSCI.3675-12.2013 23345218 PMC3711546

[B94] Zion Golumbic E. M. Ding N. Bickel S. Lakatos P. Schevon C. A. McKhann G. M. (2013). Mechanisms underlying selective neuronal tracking of attended speech at a “cocktail party”. *Neuron* 77 980–991. 10.1016/j.neuron.2012.12.037 23473326 PMC3891478

