# Supplementary figures and images for: Increases in sensory noise predict attentional disruptions to audiovisual speech perception
Source: Front Hum Neurosci. 2023 Jan 4;16:1027335. doi: 10.3389/fnhum.2022.1027335 (PMC9846366; doi:10.3389/fnhum.2022.1027335)

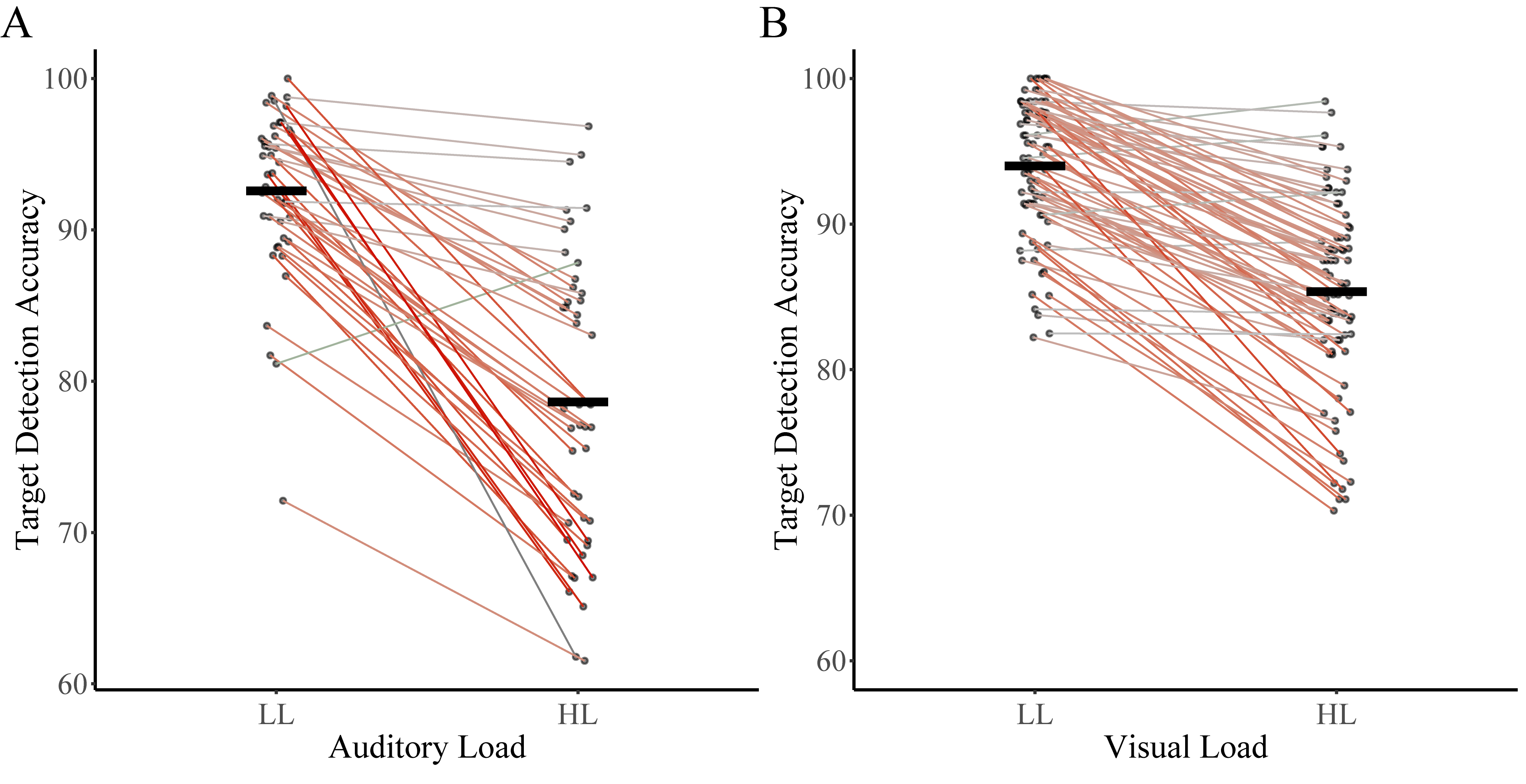

Supplement: Supplementary Figure 1 — Performance on low-load (LL) and high-load (HL) distractor tasks. Participants scored significantly higher during the LL conditions than the HL conditions for both auditory (A; LL = 92.6; HL = 78.6; t44 = 10.39, p = 2.04 × 10^–13^, Cohen’s d = 1.79) and visual (B; LL = 94.0; HL = 85.4; t76 = 13.81, p = 2.02 × 10^–22^, Cohen’s d = 1.48) distractor tasks. [file Image_1.TIF]

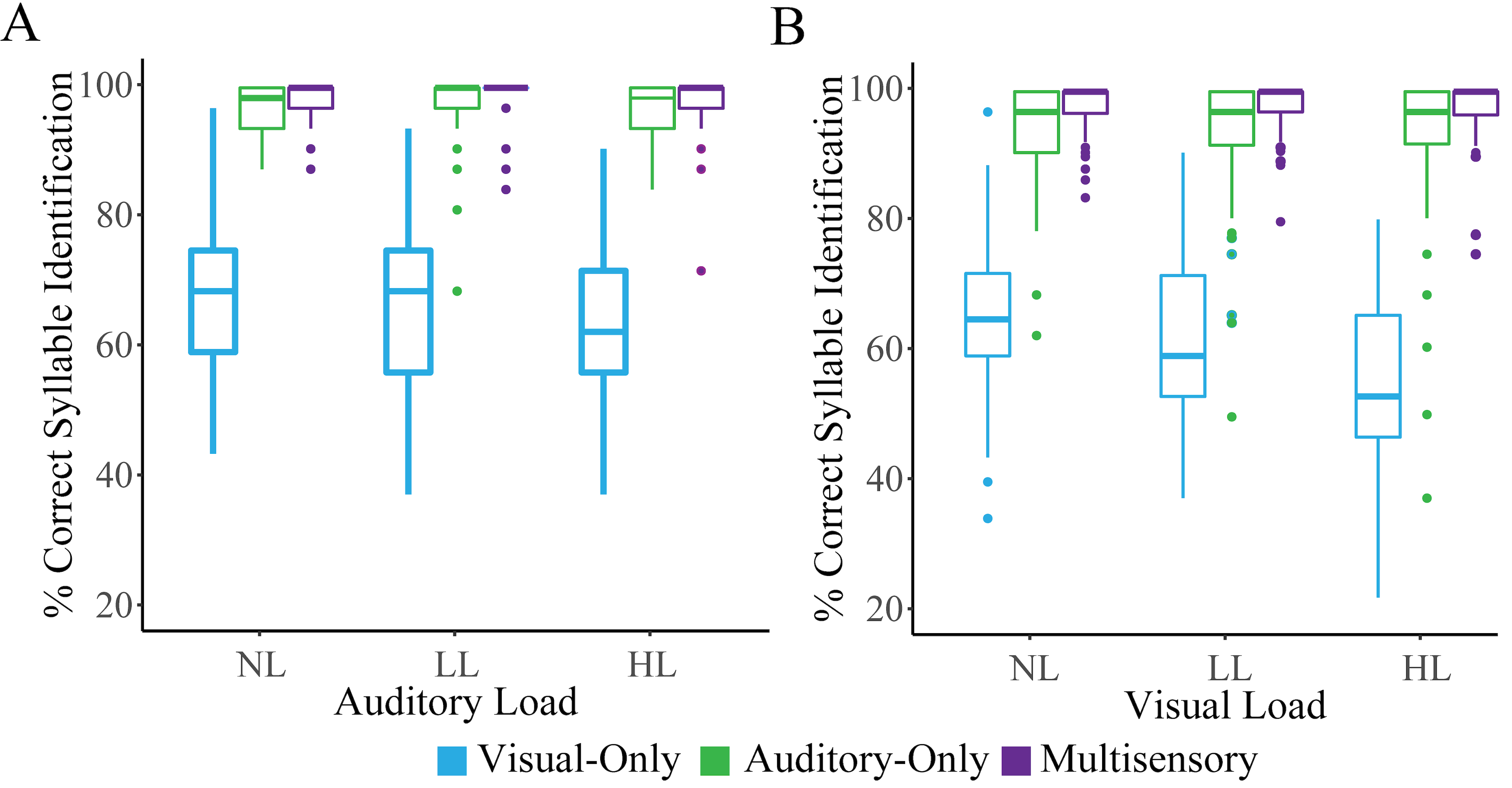

Supplement: Supplementary Figure 2 — Performance on unisensory and congruent multisensory trials for no load (NL), low load (LL), and high load (HL) blocks. Percent correct syllable identification for visual-only, auditory-only, and multisensory congruent trials for the auditory distractor (A) and visual distractor (B) tasks. An RMANOVA revealed that distractor modality (F1,128 = 12.8, p = 4.91 × 10^–4^, partial η2 = 0.091), perceptual load (F2,256 = 6.7, p = 0.001, partial η2 = 0.050), and syllable modality (F2,256 = 1040.5, p = 1.16 × 10–123, partial η2 = 0.890) significantly altered accuracy. [file Image_2.TIF]

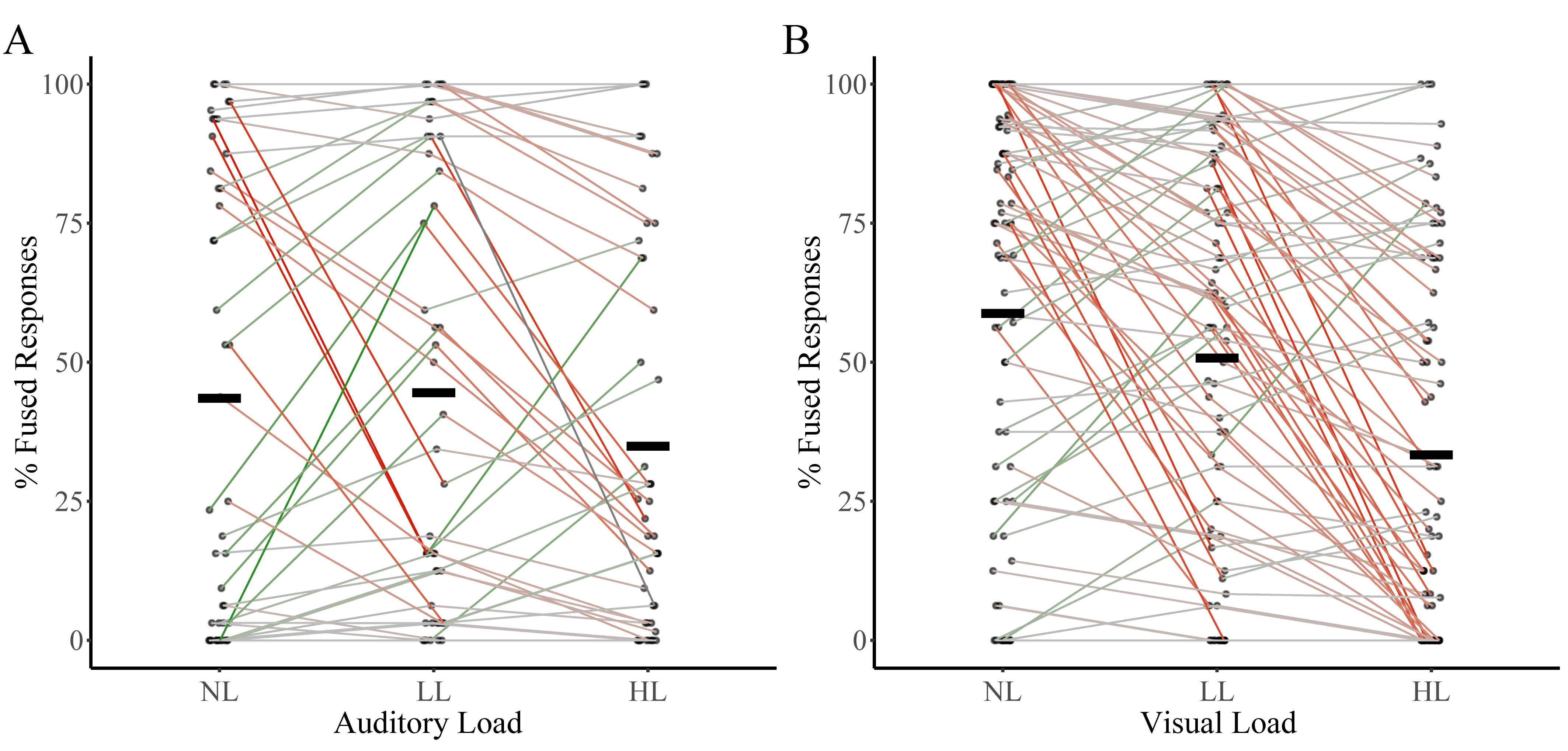

Supplement: Supplementary Figure 3 — McGurk fused reports for no load (NL), low load (LL), and high load (HL). The percent of fused reports (“da” or “tha”) during each block are shown for auditory distractor (A) and visual distractor (B) tasks. Horizontal bars indicate group averages. Colored lines connect individual percent fused reports across each block. Green lines indicate increased in fused reports and a red line indicates a decrease in fused reports. An RMANOVA revealed that both perceptual load (F2,256 = 22.5, p = 9.90 × 10^–10^, partial η2 = 0.148) and the interaction between load and distractor modality (F2,256 = 4.7, p = 0.010, partial η2 = 0.035) significantly altered percent McGurk reports. [file Image_3.TIF]
